# The Diversity of Cyanobacterial Toxins on Structural Characterization, Distribution and Identification: A Systematic Review

**DOI:** 10.3390/toxins11090530

**Published:** 2019-09-12

**Authors:** Xingde Du, Haohao Liu, Le Yuan, Yueqin Wang, Ya Ma, Rui Wang, Xinghai Chen, Michael D. Losiewicz, Hongxiang Guo, Huizhen Zhang

**Affiliations:** 1 College of Public Health, Zhengzhou University, Zhengzhou 450001, China; duxd1993@gs.zzu.edu.cn (X.D.); lhl2019@gs.zzu.edu.cn (H.L.); ylsir2018@gs.zzu.edu.cn (L.Y.); wangyueqin@gs.zzu.edu.cn (Y.W.); maya@gs.zzu.edu.cn (Y.M.); wangrui2019@gs.zzu.edu.cn (R.W.); 2 Department of Chemistry and Biochemistry, St Mary’s University, San Antonio, TX 78228, USA; xchen@stmarytx.edu (X.C.); mlosiewicz@stmarytx.edu (M.D.L.); 3 College of Life Sciences, Henan Agricultural University, Zhengzhou 450002, China

**Keywords:** cyanobacterial toxins, diversity, structural characterization, distribution, identification

## Abstract

The widespread distribution of cyanobacteria in the aquatic environment is increasing the risk of water pollution caused by cyanotoxins, which poses a serious threat to human health. However, the structural characterization, distribution and identification techniques of cyanotoxins have not been comprehensively reviewed in previous studies. This paper aims to elaborate the existing information systematically on the diversity of cyanotoxins to identify valuable research avenues. According to the chemical structure, cyanotoxins are mainly classified into cyclic peptides, alkaloids, lipopeptides, nonprotein amino acids and lipoglycans. In terms of global distribution, the amount of cyanotoxins are unbalanced in different areas. The diversity of cyanotoxins is more obviously found in many developed countries than that in undeveloped countries. Moreover, the threat of cyanotoxins has promoted the development of identification and detection technology. Many emerging methods have been developed to detect cyanotoxins in the environment. This communication provides a comprehensive review of the diversity of cyanotoxins, and the detection and identification technology was discussed. This detailed information will be a valuable resource for identifying the various types of cyanotoxins which threaten the environment of different areas. The ability to accurately identify specific cyanotoxins is an obvious and essential aspect of cyanobacterial research.

## 1. Introduction

Cyanobacteria, which have existed for about 3.5 billion years, are believed to be the oldest creatures on Earth. Even though over 2600 cyanobacterial species have been described so far, it is believed that there are still many unknown species [1]. Cyanotoxins are toxic secondary metabolites produced by cyanobacteria, and pose a major threat to the ecological environment and human health. Cyanotoxins are diverse in functional properties and chemical structures. Based on functional properties, cyanotoxins can be mainly classified into hepatotoxins, neurotoxins, dermatotoxins and cytotoxins [2]. According to the chemical structure, cyanotoxins are mainly divided into cyclic peptides, alkaloids, lipopeptides, non-protein amino acids and lipoglycans. Different chemical structures of cyanotoxins contribute to the diversity of their functional properties. A number of lipopeptide cyanotoxins have been found to have peculiar biological activities [3,4], such as anticancer, antifungal and molluscicidal activities. However, there are still many cyanotoxins that are believed to have special activities that have not yet been identified.

Cyanobacterial toxins are widely distributed in fresh and saltwater, and they have been reported hundreds of times in various countries and regions of the world, even in the Arctic and arid desert areas [5,6]. The extensiveness of the distribution of cyanotoxins is self-evident, however, the characteristics of the distribution and the severity of cyanobacterial toxins vary in different areas. For example, in the Baltic region, cyanobacterial blooms covering an area of 100,000 square kilometers can be formed in the summer [7], and various cyanotoxins produced by this cyanobacteria are seriously threatening the surrounding countries.

The harm of cyanotoxins is obvious, and it has been regarded as a new public health concern by the World Health Organization (WHO) [5]. Common methods such as bioassays, biochemical analysis, chemical assays and molecular assays have been used to detect cyanotoxins. However, because of the diversity of cyanotoxin species and chemical structure, the current routine methods cannot be used to detect all the types and variants of cyanotoxins. Therefore, the development of valuable techniques and methods of detecting cyanotoxins are prerequisite for the accurate identification of cyanobacterial toxins in the environment.

For these reasons, data on the structural characterization and regional distribution of cyanotoxins over the past twenty years were collected for this paper to provide a review of the cyanotoxin diversity in the world. We also summarize and discuss the protocols and advanced detection methods for cyanotoxins reported in recent years.

## 2. Structural Characterization

### 2.1. Cyclic Peptides

**Figure 1:**
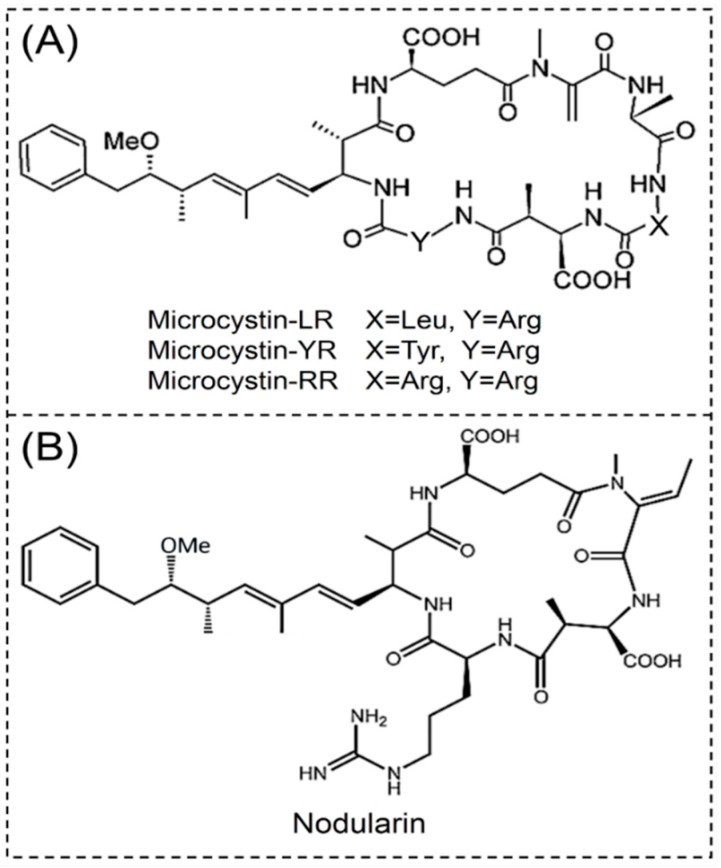
Structure of cyclic peptide cyanotoxins. (**A**) Microcystin-LR, YR and RR, (**B**) Nodularin.

A cyclic peptide is a cyclic polypeptide chain formed by a covalent linkage between the amino termini and the carboxyl termini, the amino termini and the side chain, the carboxyl termini and the side chain, or the side chain with another side chain. Microcystins (MCs) and nodularins (NODs) are two classical cyclic peptide cyanobacterial toxins (Figure 1).

#### 2.1.1. Microcystins

MCs are hepatotoxic cyclic heptapeptides. The general structure of a MC is cyclo (-D-Ala-L-X-D-*erythro*-*β*-methylAsp-L-Y-Adda-D-Glu-*N*-methyldehydro-Ala), in which Adda is the unusual C20 amino acid (3-amino-9-methoxy-2,6,8-trimethyl-10-phenyl-4,6-dienoic acid) [8]. Nuclear magnetic resonance (NMR) was used for the structure determination of MCs in the 1990s [9]. In recent years, more sensitive mass spectrometry (MS) techniques have enabled the detection of MCs in trace amounts in cyanobacterial samples. To date, 246 MC congeners have been found [10]. The most common congeners are MC-LR, MC-YR and MC-RR, resulting from the presence of the L-forms of leucine (L), tyrosine (Y) or arginine (R) in positions 2 and 4 (Figure 1A). The structure diversity of MCs results in significant differences in toxicity, and the toxicity of MCs has been demonstrated in 79 countries [11]. As one of the most frequent and toxic variant, MC-LR had been classified as a possible human carcinogen (group 2B) by the International Agency for Research on Cancer (IARC) in 2010 [12], and the WHO has established a provisional guideline of 1 µg/L MCs for human drinking water.

Investigations have found that the distribution of MCs in freshwater habitats is a matter of concern, and MCs are typically produced by several cyanobacterial genera including *Anabaena*, *Dolichospermum*, *Geitlerinema, Leptolyngbya*, *Microcystis*, *Nostoc*, *Phormidium* and *Planktothrix* [13]. In addition, MCs have also been found in many marine cyanobacteria [14] such as *Spirulina, Synechococcus* and *Trichodesmium* (Table 1).

Biochemical methods, including enzyme-linked immunosorbent assays (ELISA) and phosphatase inhibition assay (PPIA), are well suited for the quick screening of MCs in water samples [15]. However, physicochemical methods are more sensitive and accurate than biochemical analysis and can provide quantitative analysis. For example, ultraviolet (UV) absorbance detection method can detect sub-nanogram amounts of toxin, and major congeners of MCs are easily identified and quantified by reversed-phase high-performance liquid chromatography (HPLC) [16]. In recent years, ultra-HPLC (UHPLC) tandem MS becomes the preferred technique for quantitative analysis and detection of cyanotoxins in water bodies, cyanobacteria and shellfish, due to its high efficiency and high-sensitivity [17].

#### 2.1.2. Nodularins

NODs are hepatotoxic cyclic pentapeptides. The structure of NODs are similar to MCs, consisting of D-glutamic acid, N-methyldehydrobutyrine, D-*erythro*-*β*-methylaspartic acid and L-arginine (Figure 1B) [11]. Since the first description of an NOD in 1988, ten congeners have been characterized [10], and NOD-R is the most often found. NODs are considered to be liver tumor initiators and promoters [18]. According to the IARC evaluation, NODs are not classifiable to their carcinogenicity to humans, because of the lack of exposure data [12]. However, NODs are potent cyanotoxins that have been shown to be associated with death in animals and humans, and appear to be more toxic than MCs.

NODs are widely distributed throughout the temperate and subtropical regions, and are mainly found in the coastal sea water and freshwater. In the past, NODs produced by cyanobacteria had only been found in *Nodularia* [11], but the latest reports found that NODs were also isolated from the newly identified cyanobacteria *Iningainema* (*Scytonemataceae*) in Australia [19] (Table 1).

NODs and MCs are similar in structure, so it is expected that NODs have similar molecular mechanisms of toxicity to MCs [8]. Therefore, the common methods for identifying and detecting MCs are equally applicable to NODs.

### 2.2. Alkaloids

Alkaloids are basic organic compounds containing nitrogen. They vary in type and structure, so the properties of different alkaloids can greatly vary. Most alkaloids have a complex cyclic structure. Six alkaloid cyanotoxins are introduced here, including cylindrospermopsins (CYNs), saxitoxins (STXs), anatoxin-a (ATX-a), anatoxin-a(s) (ATX-a(s)), lyngbyatoxins and aplysiatoxins.

#### 2.2.1. Cylindrospermopsins

**Figure 2:**
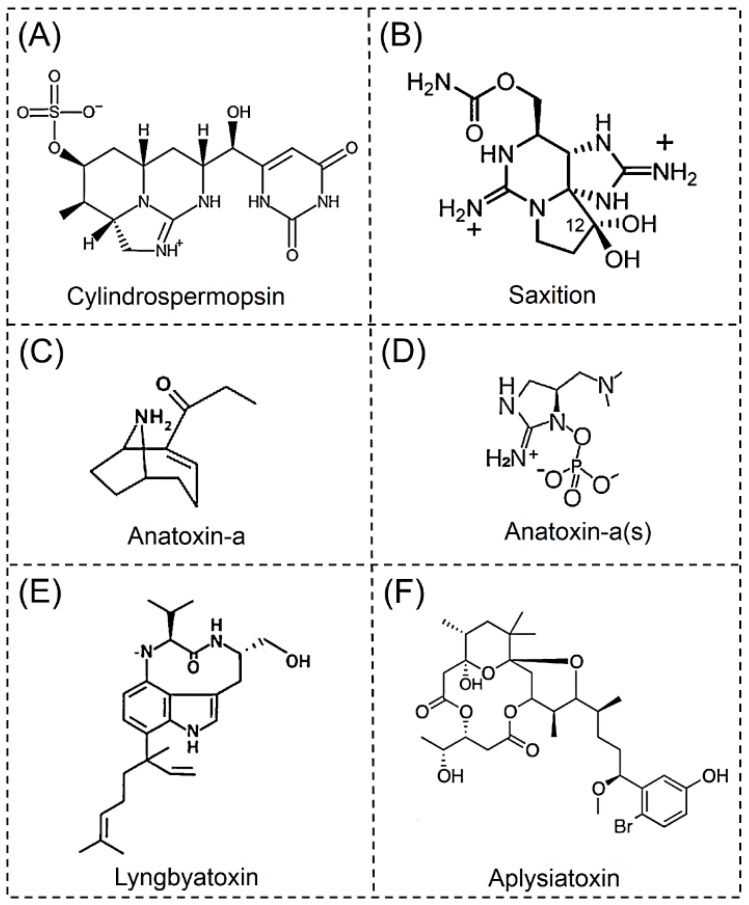
Structure of alkaloid cyanotoxins. (**A**) Cylindrospermopsin, (**B**) Saxitoxin, (**C**) Anatoxin-a, (**D**) Anatoxin-a(s), (**E**) Lyngbyatoxin, (**F**) Aplysiatoxin.

A CYN is an alkaloid hepatotoxin that is formed from a tricyclic guanidine moiety combined with hydroxymethyluracil (Figure 2A) [58]. The chemical structure of a CYN was first identified in 1992 [59]. Currently, there are five known CYN analogs, namely CYN, 7-Deoxydesulfo-CYN, 7-deoxydesulfo-12-acetyl-CYN, 7-*epi*-CYN, 7-deoxy-CYN [25]. CYNs primarily target the liver, but it is also a cytotoxin. Recent studies have found that CYNs also cause nervous system damage, and they are more toxic than MCs in terms of neurotoxicity [58,60].

CYNs were first reported in 1979 after a hepato-enteritis outbreak which was caused by a *Cylindrospermopsis* bloom in Palm Island (Queensland, Australia) [61]. CYNs are mainly found in fresh water and seawater in tropical, subtropical, and temperate climate zones. CYNs are produced by various cyanobacteria, including *Aphanizomenon*, *Chrysosporum*, *Cylindrospermopsis*, *Oscillatoria* and *Raphidiopsis* [23] (Table 1).

Reverse phase-HPLC coupled with photodiode array detection was the first screening method used for CYNs [62]. Unlike many other cyanotoxins, CYNs are often present in the form of extracellular dissolution [25]. There is little research on the detection of CYNs in drinking water and recreational water, and clearly, an effective method is crucial. Presently, the methods most widely used for the detection and identification of CYNs include bioassays, ELISA, liquid chromatography (LC), capillary electrophoresis (CE) and molecular methods [25,63]. CYNs from multiple types of samples can be quantified by ELISA with high sensitivity. However, it is nonselective for CYNs analogues and cross-reactivity may occur. LC-MS has been proven to be an ideal method for discovering trace CYNs in water samples because of its sensitivity and specificity, and it has been established as the standard method for CYNs detection [64].

#### 2.2.2. Saxitoxins

STX, a neurotoxic alkaloid, is the most representative paralytic shellfish poison. The structure of STX, which was finally confirmed in 1971, is composed of a 3,4-perhydropurine tricyclic system with two guanidinium groups (Figure 2B) [65]. Currently, 57 STXs have been described [28]. STX is one of the most lethal toxins, and it has been used in chemical weapons [66]. STXs cause an annually estimated 2000 cases of paralytic shellfish poisoning globally, with a mortality rate of 15% [67]. In reference to previous poisonings, an estimated dose of 0.5–1 mg of STX is considered toxic for humans. The Food and Drug Administration recommends that the concentration of STXs in shellfish food should not exceed 80 μg/100 g [21].

STXs are produced by cyanobacterial and accumulate in shellfish, which can then be ingested by humans. *Anabaena*, *Aphanizomenon*, *Cuspidothrix*, *Cylindrospermopsis*, *Dolichospermum*, *Fischerella* and *Geitlerinema* are some of the potentially STX-producing genera (Table 1).

Common physicochemical methods, toxicology-based biological methods, and biochemical methods are suitable for the detection and quantification of STXs [68]. The previous “gold standard” for STX measurement was the mouse bioassay, which has been refined and standardized by the Association of Official Analytical Chemists to provide quick and adequately accurate measurements. Nowadays, cytotoxicity tests have become an alternative to the mouse bioassay as a routine monitoring method to detect STXs [69]. Immunoassays based on the interactions of an antibody and its target is also commonly used for the detection of STXs. Owing to cross-reactivity within the different derivatives, it is recommended that ELISAs be used as screening tools rather than quantitative assays [68]. In the last decade, LC-MS has been successfully adapted for the analysis of STXs, and hydrophilic interaction liquid chromatography (HILIC) is often used for separation and selective reaction monitoring [70]. If only sensitivity and selectivity are considered, HPLC-fluorescence (HPLC-FL) and HPLC-MS are still the best methods [71].

#### 2.2.3. Anatoxin-a

Anatoxins (ATXs) are neurotoxins isolated from cyanobacteria *Anabaena*. ATX-a and ATX-a(s) are the two most important ATXs. ATX-a is a neurotoxic bicyclic alkaloid, containing 2-acetyl-9-aza- bicycle(4.2.1)non-2-ene (Figure 2C) [72]. ATX-a was the first cyanotoxin to be structurally resolved by X-ray crystallography and confirmed by ^1^H-NMR [72]. The four congeners of this compound are ATX-a, homoATX-a, dihydroATX-a and dihydrohomoATX-a [30]. HomoATX-a is a potent analogue of ATX-a. First synthesized in 1992, the structure has a propionyl group in place of the acetyl group [73]. ATX-a and homoATX-a are nicotinic agonists and have been shown to bind to nicotinic acetylcholine receptors [52]. In sufficiently high concentrations, ATXs can paralyze the nervous system and cause death. However, these neurotoxins are unstable and have a short half-life [74]. They can be rapidly degraded into nontoxic products, especially in light and high pH conditions.

ATX-a was first isolated from the freshwater cyanobacteria *Anabaena flos-aquae* in the 1970s. To date, ATX-a has been reported to exist in many cyanobacterial genera, such as *Anabaena, Aphanizomenon*, *Arthrospira, Cuspidothrix*, *Cylindrospermum*, *Dolichospermum*, *Oscillatoria* and *Phormidium* (Table 1).

Apart from bioassays, most reported methods for detecting ATX-a are based on chemical assays. HPLC coupled with UV or fluorescence detection, gas chromatography/Mass Spectroscop (GC/MS), and HPLC-MS are becoming the most common techniques [75]. Among them, LC-MS/MS offers perhaps the best analytical approach to determining ATX-a, homoATX-a, their degradation products and analogues [74]. Furthermore, qPCR allows the quantification of ATX-a gene *(anaC)* copy numbers in environmental samples. Recently, a new tested PCR-based method to detect ATX-a in aquatic ecosystems was developed, improving the detection level of *anaC* in environmental samples [76].

#### 2.2.4. Anatoxin-a(s)

ATX-a(s) is a unique guanidinium methyl phosphate ester that is also a neurotoxic alkaloid [77]. The structure of ATX-a(s) was determined by NMR and MS in 1989 [78], and its structure was determined to be (5*S*)-2-amino-1-((hydroxylmethoxyphosphinyl) oxy)-*N*,*N*-dimethyl-4,5-dihydro- 1*H*-imidazole-5-methanamine) (Figure 2D) [22]. To date, no analogues of ATX-a(s) have been found. ATX-a(s) is also a natural organic phosphorus compound [79]. It is similar to organophosphorus and carbamate insecticides, and it may act as a potent irreversible acetylcholinesterase inhibitor to cause toxicity. ATX-a(s) is produced only by *Anabaena* and is not found as frequently as other cyanobacterial toxins [22] (Table 1).

Few studies have been done on the analysis of ATX-a(s). Bioassays have been used to detect ATX-a(s) in the environment, but such assays are limited by several issues, such as the sensitivity and specificity of detection [80]. Because of the lack of chromophores on this organophosphorus molecule, HPLC-UV is not suitable for its detection [81]. The AChE inhibition assay is useful for detecting ATX-a(s), but other organophosphorus compounds may inhibit the activity of this enzyme, so this method is not always dependable [82]. Due to these limitations, it is more effective to combine multiple methods than to reply on a single approach. To ensure reliability, the ATX-a(s) in the samples should be reconfirmed by LC-MS/MS.

#### 2.2.5. Lyngbyatoxins

Lyngbyatoxins are dermatoxic alkaloids. Chemical and spectral data show that the lyngbyatoxin molecule is comprised of an indolactam ring, with an attached linalyl side group at C-7 (Figure 2E) [83]. So far, seven natural lyngbyatoxin analogues have been identified, namely lyngbyatoxin A-C, 12-*epi*-lyngbyatoxin A, 2-oxo-3(*R*)-hydroxy-lyngbyatoxin A, 2-oxo-3(*R*)-hydroxy- 13-N-desmethyllyngbyatoxin A and 2,3-seco-2,3-dioxolyngbyatoxin A [32,34,35]. Lyngbyatoxins are highly inflammatory and vesicatory substances derived from marine cyanobacteria [84]. In another study, lyngbyatoxins were reported as a causative agent of human skin irritation and marine turtle poisoning [83,85]. Lyngbyatoxins are also considered to be an effective tumor promoter that induces protein kinase C (PKC) activity [86].

Lyngbyatoxins were first isolated from the lipid extract of a Hawaiian shallow-water variety of *Lyngbya majuscula* in 1979 [83]. Lyngbyatoxins have thus far been detected in the blooms of *Lyngbya*, *Oscillatoria* and *Schizothrix* [32,33]. A recent study found that it was also present in the Campania coast of southern Italy [87] (Table 1).

Chemical analysis methods are often used to detect lyngbyatoxins in environmental samples, such as the liquid chromatogram-high resolution mass spectrum (LC-HRMS/MS) combined with bioinformatics analysis on molecular network [87]. Also, bioanalysis is an important method for the toxicity evaluation of lyngbyatoxins.

#### 2.2.6. Aplysiatoxins

Aplysiatoxins are considered to be dermatoxic alkaloids [77]. The molecular architecture of aplysiatoxins are bislactones of 3,4-dihydroxyvaleric acid and 4,6,6,10,12-pentamethyl-3,7,9,11,15- tetraoxy-15-phenylpentadecanoic acid (Figure 2F) [88]. Five analogues of this compound have been identified by NMR and MS, namely aplysiatoxin, debromo-aplysiatoxin, anhydro-aplysiatoxin, 3-methoxydebromo-aplysiatoxin, oscillatoxin A and 31-noroscillatoxin B [36]. Similar to lyngbyatoxins, aplysiatoxins induce contact dermatitis through the activation of PKC [23]. Aplysiatoxin and debromo-aplysiatoxin were also recently found to be potent tumor promoters in two-stage carcinogenesis in mouse skin [89]. Another study has showed that aplysiatoxin and its debrominated analogue induced expression of latent HIV-1 provirus in both cell line and primary cell models [90]. Aplysiatoxins are produced by the marine cyanobacteria *Lyngbya majuscula* in estuarine and coastal waters under tropical and subtropical climates [34] (Table 1).

Chemical analysis and bioanalysis are suitable for detecting aplysiatoxins in the environment and evaluating its toxic effects, respectively. It has been shown that LC-MS/MS-based molecular networking approach is a rapid analytical technique, which can be used to detect aplysiatoxins from environmental samples [36].

### 2.3. Lipopeptides

Lipopeptides are a class of compounds consisting of aliphatic chains and peptide chains, which have a wide range of biological activities. Most lipopeptides are derived from metabolites of microorganisms (including cyanobacteria).

**Figure 3:**
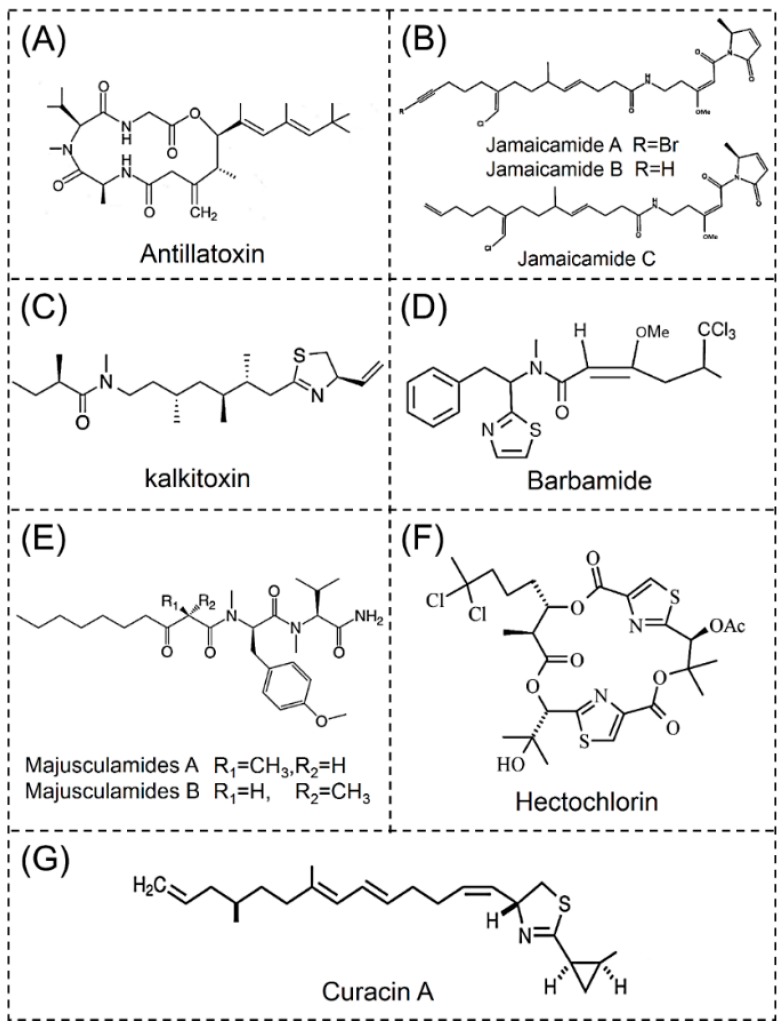
Structure of lipopeptide cyanotoxins. (**A**) Antillatoxin, (**B**) Jamaicamide A-C, (**C**) Kalkitoxin, (**D**) Barbamide, (**E**) Majusculamides A and B, (**F**) Hectochlorin, (**G**) Curacin A.

Seven lipopeptide cyanotoxins from marine cyanobacteria are described as antillatoxins, jamaicamides, kalkitoxins, barbamides, majusculamides, hectochlorin and curacins, respectively (Figure 3).

#### 2.3.1. Antillatoxins

Antillatoxin A is a neurotoxic lipopeptide that was isolated from the tropical marine cyanobacteria *Lyngbya majuscula* in Curaçao [38]. Antillatoxin B is a *N*-methylhomophenylalanine analogue also produced by *Lyngbya majuscula* that has been found in Puerto Rico and Florida [37]. The original planar structure of antillatoxins was determined by standard spectroscopic techniques. Antillatoxins are composed of a tripeptide that forms both ester and amide linkages with a highly methylated lipid section (Figure 3A), and their structural characteristics is a 9-tert-butyl-6,8- dimethyl-6,8-diene unit attached to the C5 of the cyclic peptide backbone [38,91]. Antillatoxins exhibit significant sodium channel activating and neurotoxicity that activates voltage-gated sodium channels leading to sodium influx in cerebellar granule neurons and cerebrocortical neurons [37,92]. Antillatoxins also have potent cytotoxicity toward Neuro 2a mouse neuroblastoma cells [93].

#### 2.3.2. Jamaicamides

Jamaicamides, a kind of neurotoxic lipopeptide, contain an alkynyl bromide, vinyl chloride, *β*-methoxy eneone system and pyrrolinone ring (Figure 3B). In 2004, three jamaicamides were isolated from the marine cyanobacteria *Lyngbya majuscula*, and were named as jamaicamide A-C [40]. The only difference in their structures was at the terminal of their polyketide aliphatic chains [94]. In 2015, three new analogues (Jamaicamide D-F) were discovered in the crude extract of *Lyngbya majuscula* by LC-MS and molecular networking analysis [39]. These jamaicamides have unusual biological activities, including sodium channel blocking, and arthropod and fish toxicity [23,40]. As a sodium channel blocker in neocortical neurons, jamaicamide A is approximately two to three times more potent than jamaicamide B and F [39].

#### 2.3.3. Kalkitoxins

The neurotoxic lipopeptide kalkitoxin was first isolated from the marine cyanobacteria *Lyngbya majuscula* in the Caribbean Sea [42]. Natural kalkitoxins possess a 2,4-disubstituted thiazoline, a lipophilic chain and an unsaturated CH_2_=CH_2_ unit (Figure 3C) [95]. Based on the evidence by 1D and 2D NMR spectra, six kalkitoxin isomers (3-*epi*-kalkitoxin, 7-*epi*-kalkitoxin, 8-*epi*-kalkitoxin, 10-*epi*-kalkitoxin, 10-*nor*-kalkitoxin and 16-*nor*-kalkitoxin) have been synthesized [41]. Kalkitoxins are strongly ichthyotoxic, and toxic to brine shrimp. They inhibit cell division, suppress inflammation and potently block voltage-sensitive sodium channels in murine neuro-2a cells [42]. In humans, Kalkitoxins have a neurotoxicity that acts on sodium channels and mitochondria [96].

#### 2.3.4. Barbamides

Barbamide is a recently discovered lipopeptide with molluscicidal activity and it is isolated from the marine cyanobacteria *Lyngbya majuscula* [97]. The structure of barbamide was determined by spectroscopic methods, and the several unique structural features include a trichloromethyl group and the methyl enol ether of a *β*-keto amide (Figure 3D) [98]. Heterologous expression of the barbamide biosynthetic gene cluster resulted in the production of a new barbamide analogue named as 4-O-demethylbarbamide [43]. This new compound is several-fold more potent than barbamide as a molluscicide. If it lacks other toxicities, it may be a superb candidate for treating snail-infested waterways which pose health risks for human populations [43].

#### 2.3.5. Majusculamides

There are four Majusculamides (majusculamides A-D) that have been found. Majusculamides A and B are cytotoxic lipopeptides isolated from the marine cyanobacteria *Lyngbya majuscula,* with antilarval settlement activities [31,34]. Their structures were elucidated using high-resolution fast atom bombardment mass spectrometry (HR-FAB-MS), and 1D and 2D NMR analyses [34]. Majusculamide A was identified as N-((2*R*)-2-methyl-3-oxodecanoyl)-D-N,O-dimethyltyrosyl-L- N-methylvalinamide [45]. Majusculamide B is an epimer of majusculamide A (Figure 3E). Majusculamide C was previously found in cyanobacteria from the Marshall Islands and was reported to have antifungal activity [45]. Majusculamide D was also originally isolated from *Lyngbya majuscula*. The absolute configuration of the 1,3-dimethyloctanamide motif was determined by the synthesis of this fragment via zirconium-catalyzed asymmetric carboalumination chemistry [44]. Majusculamides possess potent cancer cell toxicity, exhibiting selective cytotoxicity toward both pancreatic (PANC-1) and glioblastoma (U251N) cancer cell lines [44].

#### 2.3.6. Hectochlorins

Hectochlorin is a cytotoxic lipopeptide isolated from marine cyanobacteria *Lyngbya majuscula* in Jamaica and Panama [99]. The planar structure of this compound was determined by 1D and 2D spectroscopy, and X-ray crystallography was used to determine the absolute stereochemistry. Hectochlorin resembles lyngbyatoxin in structure (Figure 3F) [47]. In 2015, three hectochlorin analogs were identified from mass spectroscoscopy based-molecular networking, respectively (Hectochlorin B-D) [39]. Hectochlorin has the ability to promote actin polymerization and shows cytotoxicity and potent inhibitory activity toward the fungus *Candida albican* [47,99]. Hectochlorin has also shown great potency against several cancer cell lines, namely colon, melanoma, ovary, and kidney [100]. Deacetylhectochlorin, a derivative of hectochlorin, was isolated from the Thai sea hare (*Bursatella leachii*). This cognate is also a potent stimulator of actin assembly, and exhibits more potent toxicity than hectochlorin against several human carcinoma cell lines [47]. The artificial synthesis of hectochlorin is the focus of current researches [99,101].

#### 2.3.7. Curacins

Curacin A is a marine cytotoxic lipopeptide found in strains of the tropical marine cyanobacteria *Lyngbya majuscula*, in Curacao [48]. NMR analysis showed that its structure contains one cysteine, ten acetate units and two S-adenosyl methionine-derived methyl groups (Figure 3G). There are four isomers of curacin A with similar biological activity and structure, namely (curacin B-E). Curacins B and C were isolated from a Curagao collection of *Lyngbya majuscula* [49]. Curacin D was found in *Caldora penicillata* and *Lyngbya majuscula* from the Pacific islands and the Virgin Islands. The structural elucidation of curacin D was accomplished through multidimensional NMR and GC/MS [50,51]. Curacin E was isolated from *Ophiocoma scolopendrina* at Kabira Reef on Ishigaki Island. NMR data and the GC spectrum showed that curacin E has an ethylcarbonyl terminus in its side chain [102]. Curacins exert their potent cell toxicity through interaction with the colchicine drug binding site on microtubules [103,104], and it is also a potent cancer cell toxin and antimitotic agent of cyanobacteria origin [105].

### 2.4. Nonprotein Amino Acids

**Figure 4:**
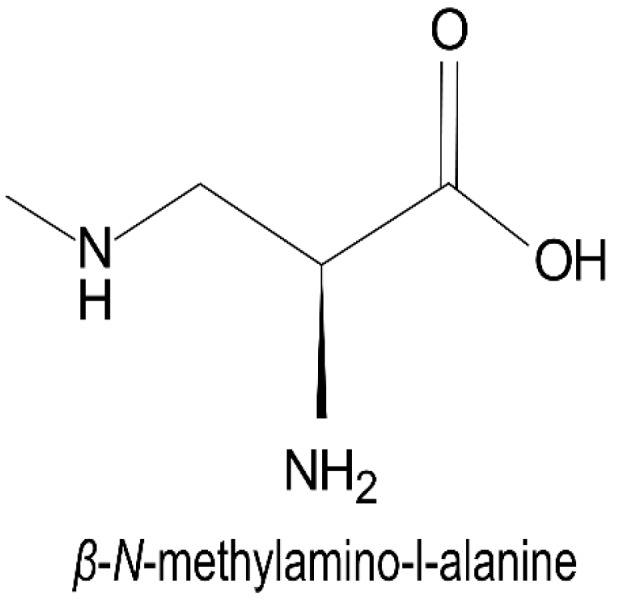
Structure of *β*-*N*-methylamino-L-alanine.

Nonprotein amino acids are natural compounds containing amino and carboxyl groups which do not constitute proteins. They are important bioactive substances mainly in microorganisms and plants. *β*-*N*-methylamino-L-alanine (BMAA, Figure 4) produced by cyanobacteria, is a type of nonprotein amino acids that was found to be a cyanotoxin. BMAA is composed of carbon with a carboxyl group, an amino group and a methylamino side chain [11,106]. Three biologically relevant structural isomers of BMAA have been identified [53], including 2,4-diaminobutyric acid (2,4-DAB), *N*-2-aminoethylglycine (AEG) and *β*-amino-*N*-methyl lanine (BAMA).

Research on BMAA has intensively increased over the past decade, and now BMAA appears to be a cause of the Guamanian ALS and Parkinsonism Dementia Complex (PDC) [107]. BMAA was first isolated from a cycad on the island of Guam in 1967. Symbiotic cyanobacteria produces BMAA in the coralloid root of the cycad, and then BMAA is transferred to and accumulated in the seeds. BMAA was once believed to occur only in cycads. However, it is now known to be produced by a range of cyanobacteria, diatoms and dinoflagellates across the world in freshwater, saltwater and terrestrial ecosystems [108,109]. BMAA is typically detected in strain cultures of cyanobacterial species belonging to *Anabaena*, *Calothrix*, *Microscystis*, *Nostoc, Scytonema*, *Synechococcus* and *Trichodesmium* [52] (Table 1).

The analysis of BMAA in native form is complicated, because of the physicochemical properties and the absence of a chromophore or fluorophore. An HPLC-FL method was developed and used for amino acid analysis in 2003 [110]. In recent years, more specific methods have been developed that have been used for BMAA analysis, such as amino acid analyzers, UPLC-UV, UPLC-MS and LC-MS/MS [111,112]. Amino acid analyzers are often used in hospital settings for analysis of amino acid contents in physiological fluids [113]. Presently, LC-MS/MS method is the most widely used analytical method to determine the content of BMAA sensitively and accurately [111]. CE-MS/MS has been developed as an alternative method for the quantitative determination of free BMAA [114]. This method displays excellent resolution of amino acid isomers and shows no interference from matrix components.

### 2.5. Lipoglycans

Lipopolysaccharides (LPS), also known as lipoglycans and endotoxin, are large molecules consisting of a lipid and a polysaccharide. LPS are major components of cyanobacterial cell walls and cover 75% of the surface area of the outer membrane [57]. In general, the fatty acid component (lipid-A) of LPS is responsible for its toxic actions. It is an irritant and can trigger allergenic responses in human and animal tissues [115].

**Figure 5:**
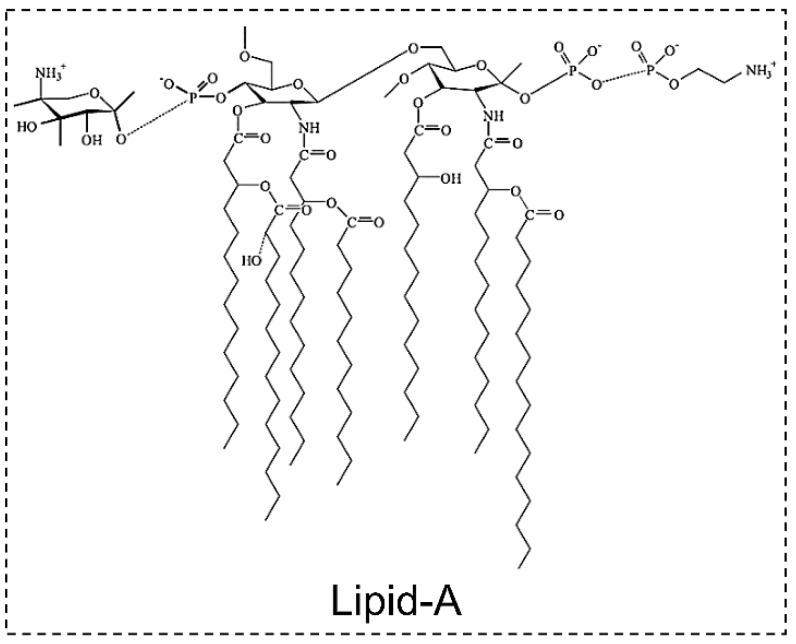
Structure of Lipid-A. Reproduced from [115]. Copyright 2005, Elsevier.

The structure of cyanobacterial LPS was determined by NMR, MS, GC and matrix-assisted laser desorption/ionization (MALDI)-MS [116]. The structure of LPS consist of an internal acylated glycolipid (lipid-A), a core domain (an oligosaccharide), and an outer polysaccharide chain (O-antigen) (Figure 5).

The toxicity of cyanobacterial LPS have been found to be less toxic than those from heterotrophic bacteria. It has been suggested that cyanobacterial LPS may cause or contribute to human illness, particularly in causing epidermal allergic reactions [116]. However, there is insufficient evidence in the scientific literature to confirm that skin contact with cyanobacterial LPS causes a skin rash. It is worth noting that cyanobacterial LPS are indirectly involved in various human diseases, including skin diseases, gastrointestinal issues, respiratory diseases, fever, allergies and headaches [117]. LPS have been shown to modulate biotransformation and toxicity of cyanotoxins and may worsen the hepatic damage induced by hepatotoxins [118].

LPS are believed to be produced by all cyanobacteria and gram-negative bacteria [57]. The production of this toxin has been reported in several cyanobacteria such as *Agmenellum*, *Anabaena*, *Anacystis*, *Microcystis*, *Oscillatoria*, *Phormidium*, *Schizothrix* and *Spirulina* [116].

Various LPS have a similar mode of biological action (toxicity) but have diverse chemical structures in different cyanobacterial strains [119]. Therefore, traditional methods such as chromatography are often not used in the analysis of LPS. Isolated LPS have been characterized and analyzed by other approaches, such as the in vivo toxicity test, the standardized pyrogenicity test with Limulus amoebocyte lysate, the advanced pyrogenicity test using the PyroGene rFC endotoxin system, or by testing the potency to activate human leukocytes [120,121]. In a recent study, a porous silicon membrane (pSiM)-based electrochemical biosensor was developed for a direct and sensitive detection of LPS [122].

## 3. Distribution of Cyanotoxins

**Figure 6:**
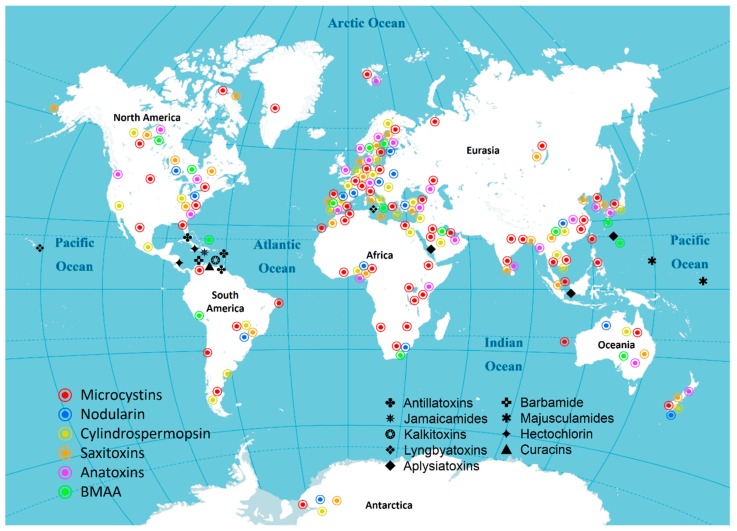
Distribution of cyanotoxins.

The global distribution of cyanobacteria toxins is presented in Figure 6. Common cyanotoxins (MCs, NODs, CYNs, STXs, ATXs and BMAA) are found in every continent except Antarctica (no BMAA and ATXs have been found in Antarctica). However, cyanotoxins are more frequently reported in Europe and North America. There are fewer reports from Asia, South America and Africa. Marine cyanotoxins are mainly distributed in the western Atlantic and central Pacific oceans.

### 3.1. Asia

Six common cyanobacterial toxins (MCs, CYNs, NODs, STXs, ATXs and BMAA) have been found in Asia (Table 2). These cyanotoxins were mainly distributed in temperate and tropical coastal areas of Asia, as well as inland lakes and rivers in various countries. Among them, MCs are the most widely distributed, which have been reported in China [123,124,125,126], Japan [127], Korea [128], Bangladesh [129], Singapore [130], Saudi Arabia [131], India [132], Philippines [133], Thailand [134], Vietnam [135,136], Israel [137] and Turkey [138]. CYNs and their homologues were mainly distributed in China [125,139], Japan [140], Saudi Arabia [141], Vietnam [142], Thailand [134,143], Israel [144] and Turkey [145]. NODs were reported only in China [146] and Turkey [138]. STXs were reported in China [147], Korea [148], India [149], Singapore [150], Bangladesh [151] and Turkey [152]. ATXs have been found in Korea [153], Qatar [154], India [149], China, Japan and Turkey [138]. And BMAA was reported in China [155], Japan [156] and Qatar [111,157].

### 3.2. Africa

There are six common cyanobacterial toxins found in Africa: MCs, NODs, CYNs, STXs, BMAA and ATXs (Table 2). Various cyanobacterial toxins have been reported in inland rivers, and in lakes in Eastern Africa, as well as coastal areas in the north. South Africa and Nigeria have also been threatened by cyanobacterial toxins. MCs were reported in Ethiopia [158], Algeria [159], Tanzania [160], Kenya [161], South Africa [162], Tunisia [163], Zimbabwe [164], Morocco [165], Uganda [166], Egypt [167], Nigeria [168] and Ghana [169]. CYNs were found in Egypt [170] and Nigeria [171]. NODs were reported in South Africa [172] and Nigeria [171]. Besides, MCs, NODs and CYNs were also found in several riverside countries along the Limpopo River [173]. STXs were reported in Morocco [174] and Nigeria [171]. ATXs were distributed in Kenya [175] and Nigeria [171]. And BMAA was only reported in South Africa [176].

### 3.3. North America

Six common cyanobacterial toxins were distributed in North America: MCs, NODs, CYNs, STXs, BMAA and ATXs (Table 2). These cyanobacterial toxins were widely distributed in inland freshwater lakes and coastal areas in the US [177,178,179,180,181] and Canada [182,183,184]. MCs [185], CYNs [186] and STXs [187] have been reported in Mexico. Also, MCs and NODs have been found in Greenland and Alaska in the arctic region [188,189].

### 3.4. South America

Although South America has had fewer reports of cyanobacterial toxins compared with other continents, five common types have been reported: MCs, CYNs, NODs, STXs, and ATXs (Table 2). In this region, Brazil is the most affected country, and all five of these toxins have been reported to be found in freshwater [190,191,192,193]. In addition, MCs were reported in Argentina [194], Chile [195] and Uruguay [196]. CYNs were also found in Argentina [194] and Uruguay [196]. Presently, BMAA has only been reported in Peru [197].

### 3.5. Antarctica

Four common cyanobacterial toxins have been found in Antarctica (Table 2). The cold climate in this area of the world is not suitable for animal survival. However, in recent years, MCs [198,199], NODs [6], CYNs [200] and STXs [201] have been described in the Antarctic. This illustrates the strong adaptability and wide distribution of cyanobacterial toxins. However, ATXs and BMAA have not been reported in Antarctica.

### 3.6. Europe

Europe is the continent with the most reports of cyanobacterial toxins, which have been found in more than 20 countries and regions (Table 2). In particular, the Baltic Sea region is highly affected by cyanobacterial toxins, and cyanobacteria form periodic blooms in the summer, covering an area of over 100,000 square kilometers [202]. A variety of cyanobacterial toxins are produced in water, posing a major threat to the survival environment of humans and animals. Six common cyanobacterial toxins were distributed in Europe. MCs were reported in Finland [203,204], Austria [205], Greece [206], Poland [207], Ireland [208], Portugal [209], Hungary [210], Norway [211], Netherlands [212], Czech [213], Germany [214], Sweden [215], Switzerland [205], Spain [216], Poland [217], the UK [218], Bulgaria [219], Romania [220], France [221,222], Serbia [223], Russia [224,225,226] and Latvia [227]. CYNs were distributed in Ireland [228], Germany [229], France [230], Italy [231], Czech [232], Spain [233], Lebanon [234], Greece [235], Finland [236], Poland [237], Sweden [215], Serbia [238], Portugal [239] and Hungary [240]. ATXs were found in Ireland [241], Italy [242], France [243], Netherlands [244], Denmark [245], Bulgaria [219], Finland [204], Poland [246], Sweden [215], Portugal [220], Spain [247], the UK [248] and Germany [249]. STXs were reported in Portugal [250], Denmark [251], France [252], Germany [253], Czech [213], Italy [254], Finland [255], the UK [256], Poland [257], Bulgaria [219], Serbia [223], Greece [235], Russia [27], Norway [258] and Spain [258]. NODs were found in Latvia [227], Germany [228], Spain [259] and France [260]. BMAA was reported in Finland [261], Sweden [262], Portugal [263], Italy [264], Poland [265], the UK [266] and France [267].

### 3.7. Oceania

Six common cyanobacterial toxins have been found in Australia (Table 2), which were detected in inland lakes and rivers of Oceania: MCs, NODs, CYNs, ATXs, STXs and BMAA [30,268,269]. The distribution of these toxins were mainly in the eastern and southern regions of Australia. These toxins have also been detected in the surrounding islands and oceans of Oceania, such as New Zealand [250,270,271,272].

### 3.8. The oceans

More than twelve cyanobacterial toxins have been found in the oceans across the world. MCs have been found in the marine environments of the central Atlantic coast of Portugal, the Canary Islands, the Brazilian coast, the Amvrakikos Gulf, and the Indian Ocean [14]. BMAA was reported from Guam in the western Pacific, and Bermuda in the western region of North Atlantic [52]. Aplysiatoxins have been reported in the Red Sea region [34], and the coast of Japan and Singapore [36,89]. Lyngbyatoxins were found in the coast of Hawaii [83] and Campania [87]. Seven lipopeptide cyanotoxins have been reported in the Caribbean and Central Pacific.

## 4. Identification of Cyanotoxins

**Figure 7:**
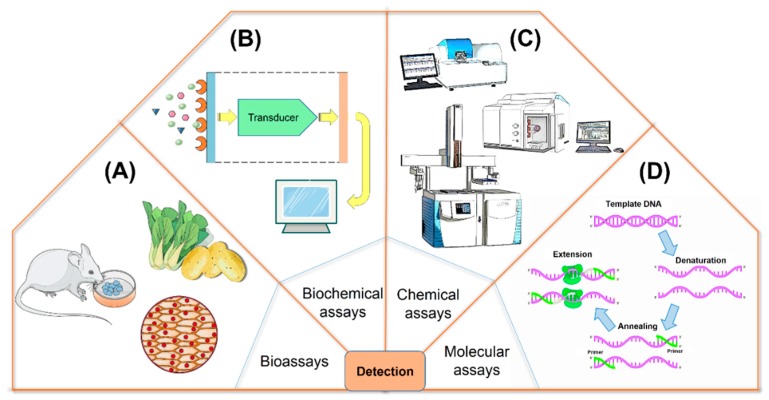
Several representative analysis techniques of cyanotoxins. (**A**) Bioassays, (**B**) Biochemical assays, (**C**) Chemical assays, (**D**) Molecular assays.

Various analytical methods have been available to detect and identify cyanobacterial toxins, such as bioassays, biochemical assays, chemical assays and molecular analysis (Figure 7A–D).

### 4.1. Bioassays

Bioassays, involving cells, plants, or animals, are an important method for monitoring the presences of cyanotoxins in environmental samples, and evaluating their effects [80].

The mouse bioassay is a common method for identifying cyanotoxins and the toxin efficacy, which is evaluated by the LD_50_ obtained from the survival time of infected animals. However, alternative chemistry and functional analysis are replacing animal trials due to ethical considerations, prolonged processes, high cost, low sensitivity and non-specificity [273]. Cytotoxicity tests have overcome the shortcomings of specificity and sensitivity in animal tests, and can provide qualitative and quantitative analysis of cyanotoxins. Immortalized cell lines and primary cells from animal tissues have been used to evaluate cyanotoxins and cyanobacterial extracts [274]. Primary cells can better reflect the physiological status in vivo than immortal cell lines. Plant tests have been used to determine the toxic effects of cyanotoxins [275]. Some plants have certain sensitivities to cyanotoxins and their physiological indicators may be affected by toxicitiy. The phytotoxic effects of MCs have been investigated by plant bioassays, and it was found that MCs can inhibite the growth and chlorophyll content of *Solanum tuberosum* culture [276]. Bioassays can provide unique information about toxic effects, which are clearly not achievable through physicochemical analysis methods. If the two methods are combined, a clear causal link can be established between the presence of a toxin and its toxic effects.

### 4.2. Biochemical Assays

Biochemical assays are detection methods that rely on the interactions between cyanotoxins and biological macromolecules. Such examples would include immunoassays, enzyme inhibition assays, and receptor bioassays. Immunoassays such as the ELISA are used to identify specific toxins in samples through antigen-antibody binding. The PPIA is a method for determining cyanotoxins by measuring protein phosphatase activity. The Receptor Assay is a method based on competitive binding between a class of neurotoxins and a receptor.

#### 4.2.1. Immunoassays

In recent years, besides the ELISA, a significant amount of antibody-based immunoassays have been developed for the detection of cyanotoxins, including Fluorescence Immunoassay (FIA) [146], immunochromatography (ICA) [277,278] and biosensor techniques [279].

The ELISA is a conventional method for the analysis of cyanotoxins, and has been used to detect MCs, NODs, CYNs, ATXs and STXs in samples [273,280]. ELISA assays provide semi-quantitative estimates of toxin concentrations. It is easy to perform an ELISA, which does not require highly skilled personnel, and it is relatively inexpensive. However, the individual variants of the toxins have not been able to be identified by ELISA, and the quantification of toxins may not be as accurate as physicochemical methods [15].

FIA is a sensitive technique for the measurement of drugs, hormones, proteins and other compounds [281]. It is often used for the quantification of cyanotoxins. Fluorescence Polarization Immunoassay (FPIA) is an immunochemical method based on FIA which can precisely quantify a target and meet the requirements of a reliable and economical screening technology [282]. The principle of FPIA is based on the difference in fluorescence polarization of the labeled antigen and labeled analyte-antibody complex [283]. Over the past decade, FPIA has been widely used in high-throughput screening of various chemical contaminants. Compared with UPLC-MS and ELISA, FPIA can detect MCs in water more simply and efficiently. The method can be extended to detect other types of cyanotoxins in the environment. However, FPIA is susceptible to interference from light scattering and endogenous fluorophores in biological samples and also from the tracer binding to sample matrix components [283].

As a combination of chromatography and an immunochemical reaction, ICA is known as a lateral flow device or strip test. It has been widely developed for point-of-care testing because of its user-friendly formats, low cost and short assay times [284]. Lateral Flow Enzyme Immunochromatography assay (LFICA) coupled with molecular imprinting technique was developed for rapid detection of MC-LR in water products [277]. However, compared with other detection methods, the significantly lower analytical sensitivity hinders its further application. A way to enhance the performance of LFICA is to eliminate the interferences from complicated matrixes. Using molecularly imprinted polymers (MIPs) as adsorbents for solid-phase extraction (SPE) columns, most of the interference in the matrix can be eliminated and sensitivity can be improved [285]. This approach achieves excellent sensitivity and specificity as well as a very low limit of detection, and it can be used to analyze other toxic components in foods and aquatic products by replacing specific MIPs and antibodies.

Biosensors are broadly used in environmental monitoring and have replaced the traditional methods of detecting toxins. Biosensors are analytical devices for the detection of an analyte, and they can provide selective quantitative or semi-quantitative analytical information. Biosensors which consist of a bio-recognition element (bioreceptor) and a transducer, can transform physiochemical information into a visual signal which can be captured by a detector [279]. The bioreceptor, can be a cell receptor, an enzyme, an organelle, etc., that binds an analyte under study. The bioreceptor is combined with the physiochemical detector or “transducer” that produces a physiochemical signal when the biosensor binds to the analyte [286]. Electrochemical and optical biosensors have been shown to detect aquatic toxins. Advanced portable biosensors would allow immediate assessment of water bodies and water treatment deficiencies [287]. Due to the simple and sensitive nature of biosensors, they can be used as an early warning monitoring tool for semi-quantitative screening of possible biotoxins in water samples.

#### 4.2.2. Enzyme Inhibition Assays

PPIA is a common enzyme inhibition assay used in the detection of cyanotoxins. Protein phosphatases are highly conserved in eukaryotes and are involved in many cellular processes [288]. A number of compounds are known to inhibit protein phosphatases, including MCs and NODs from cyanobacteria [288,289]. Cyanotoxins can be detected by following the degree of inhibition of protein phosphatases. The PPIA can not only identify a toxin, but also estimate its toxicity. PPIA has good sensitivity and does not seem to be affected by sample matrices, except for very high concentration of cellular extracts [290].

#### 4.2.3. Receptor Bioassays

The Receptor Bioassay has become the most recognized method for the detection and quantification of ATXs and STXs in shellfish and water [291]. The principle of this approach is based on the mechanism of certain toxins that compete with the nicotinic acetylcholine receptor. However, nicotinic acetylcholine receptors can also bind with spirolides, gymnodimines and other marine toxins [273,292]. Therefore, this method cannot distinguish the specific type of toxin. Solving this problem will involve the designing of specific and sensitive cholinesterase receptors.

### 4.3. Chemical Assays

Chemical analysis is the most reliable technique for detecting and identifying cyanobacterial toxins in samples based on the physical and chemical properties of cyantoxins. Some advanced chemical analysis methods have been used to detect and identify cyanobacterial toxins accurately and sensitively [293], such as NMR, HPLC, CE, LC-MS, GC, etc. Another technique is matrix-assisted laser desorption-ionization-time-of-flight mass spectrometry (MALDI-TOF-MS), which can distinguish toxic from non-toxic strains at the level of a single colony or filament, without prior solvent extraction [294,295]. Currently, MS-based methods are considered to be the best approach to unambiguously identify and quantify the different variants of cyanotoxins. However, they are not suitable for site testing because these analytical methods often require harmful solvents, expensive instruments, skilled personnel, and complex operational procedures. They are also time consuming and typically subjected to high cost [273,296].

### 4.4. Molecular Assays

Molecular assays based on the polymerase chain reaction (PCR) have been developed over the past 20 years. The sequencing of cyanobacterial genomes has led to the identification of the gene clusters responsible for cyanotoxin production, which paved the way for the use of these genes as targets for PCR [297]. Three different PCR-based methods have been used for the detection of cyanotoxins [298,299,300]. The first approach is based on conventional PCR targeting only one gene. This method provides qualitative results that can be used to detect potential cyanobacterial toxins at the beginning of the bloom. In order to get a quantitative estimation of cyanotoxin gene abundance, the second approach was developed, which is based on the use of quantitative PCR (qPCR) targeting a gene involved in the biosynthesis of cyanotoxins. Finally, the combination of PCR and DNA microarray can be used to detect potentially toxic cyanobacterial species or to identify specific genes involved in the biosynthesis of cyanobacterial toxins [301]. However, it may be found that the actual toxin concentration in samples is inconsistent with qPCR results, which may be caused by various technical reasons and biological problems [286]. Therefore, the better choice to evaluate cyanotoxins was combining traditional analytical methods and PCR techniques.

Recently, microarray technologies have been used to achieve simultaneous detection and semi-quantification of cyanotoxins in environmental samples [302]. A detection method based on microsphere array coupled flow cytometry was developed [303]. The process can be used as a semi-quantitative screening tool for cyanobacterial toxins in fresh or brackish water. This method can save time and greatly reduce sample size due to the simultaneous detection of multiple cyanotoxins.

Fluorescence in Situ Hybridization (FISH), uses a fluorescein-labeled oligonucleotide probe to hybridize with nucleic acid target sequences in samples, to obtain specific information on gene expression. In FISH imaging, specific rRNA probes allow the identification of microorganisms at the genus level, while mRNA probes will give information on the expression of a particular gene [304]. Therefore, FISH imaging might be a very powerful technique for the detection and identification of toxic cyanobacteria, particularly those that produce MCs in the environment [304,305,306]. Some species in suboptimal growth conditions may have lower mRNA levels, in which case the sensitivity of the assay can be increased by Tyramine Signal Amplification (TSA-FISH) [307,308]. This supplement to the method involves a specific oligonucleotide that is linked to horseradish peroxidase (HRP), which catalyzes the permanent deposition of many fluorescent tyramides in the probe surroundings. This technique showed 10 to 20 fold signal amplifications relative to fluorescein-monolabeled probes [308,309,310]. For all these reasons, TSA amplification is highly recommended for the microscopic detection of planktonic algae.

These methods look very promising, but so far their application remains limited to the research level [298]. And the cost of these methods still hampers their use in monitoring applications.

## 5. Final Remarks

The diversity of cyanobacteria leads to a variety of cyanobacterial toxins. According to the chemical structure, cyanobacterial toxins are mainly classified into cyclic peptides, alkaloids, lipopeptides, nonprotein amino acids and lipoglycans. The producing genera of cyanotoxins have been summarized in Table 1. MCs, CYNs, ATXs, STXs and BMAA can be produced by a variety of cyanobacteria. NODs are only produced by *Anabaena*. LPS exists in the cell walls of almost all cyanobacteria [57]. Lipopeptide cyanotoxins are mainly produced by the marine cyanobacteria *Lyngbya majuscula*.

We reviewed the distribution of cyanobacterial toxins around the world (Figure 6, Table 2). MCs were found to be the most widely distributed cyanotoxins (57 out of 60 countries), followed by CYNs (31 out of 60), STXs (29 out of 60), ATXs (26 out of 60), BMAA (16 out of 60) and NODs (13 out of 60). It was found that the diversity of cyanotoxins is mainly reflected in developed countries. The reports of cyanotoxins in developed areas are far more than that in underdeveloped areas. More than four cyanobacterial toxins were reported in 18 countries (China, the US, Canada, France, Australia, New Zealand, Turkey, Nigeria, Brazil, Finland, Germany, Italy, Sweden, Poland, Portugal, Spain, Japan and the UK). Of these 18 countries, 13 are developed regions, accounting for 72%. Furthermore, there are 15 countries where only one cyanotoxin was reported and 14 of which are developing countries (except Switzerland). However, we are more inclined to believe that cyanotoxins may have a greater threat in less-developed areas due to the possible lack of monitoring activities in these areas.

There are many available methods to detect and identify cyanobacterial toxins, such as bioassays, biochemical assays, chemical assays and molecular analysis. At present, there is no single method that is the optimal for the detection and identification of all types of cyanobacterial toxins, and each method has its applicability [311]. Detection methods are affected by the variety and abundance of cyanotoxins. The choice of method is also inevitably influenced by the availability of the analytical equipment and its applicability in a particular environment. For example, molecular methods are more suitable for detecting potential toxic cyanobacteria, and immune sensors are better at monitoring the presence of toxins in samples and bioassays are often used to evaluate toxic effects. Thus, research purpose, economic feasibility, speed of analysis, sensitivity and field applicability should be taken into account when selecting detection methods. Previous experience shows that combining multiple methods can greatly improve the detection efficiency. It is still necessary to develop new techniques for detecting and identifying cyanobacterial toxins more easily and sensitively. Currently, plants have become an area of interest in many studies. It was found that plants can respond to other plants and animals in the environment [312]. Perhaps plants can be used as alternatives to animal experiments in the future after people have a deeper understanding of them.

Cyanobacterial toxins are widely distributed and varied, and their harmfulness cannot be ignored. Epidemiological studies and animal experiments have confirmed that many cyanotoxins have multi-organ toxicity and carcinogenic effects, posing a serious threat to human health and life quality [22,313]. To reduce the threat of cyanotoxins to humans, it is essential to strengthen the monitoring of cyanobacterial toxins worldwide, especially in underdeveloped areas. In recent years, cyanotoxins with special biological activities have attracted wide attention. Most of these cyanotoxins are produced by marine cyanobacteria. It was found that the lipopeptide cyanotoxins not only have neurotoxicity and cytotoxicity but also have anticancer, antifungal and molluscicidal activities [3]. These specific types of natural toxins have unique pharmacological properties and show great potential of being developed and utilized for human disease. Marine cyanobacteria are still a rich source of untapped natural products. However, the exploration of marine cyanotoxins is just beginning.

## Figures and Tables

**Table 1:** Category, common name, primary toxicity, analogues and producing genera of cyanotoxins.

Category	Common Name	Primary Toxicity	Analogues	Producing Genera	Reference
**Cyclic peptides**	Microcystins	Hepatotoxicity	246	*Anabaena, Aphanizomenon, Dolichospermum, Fischerella, Geitlerinema, Hapalosiphon, Leptolyngbya, Limnothrix, Merismopedia, Microcystis, Nostoc, Oscillatoria, Phormidium, Planktothrix, Pseudanabaena, Radiocystis, Pseudoanabaena, Spirulina, Synechococcus, Trichodesmium, Woronichinia*	[6,11,13,14,20,21,22]
	Nodularins	Hepatotoxicity	10	*Iningainema, Nodularia*	[10,11,19]
**Alkaloids**	Cylindrospermopsins	Hepatotoxicity	5	*Anabaena, Aphanizomenon, Chrysosporum, Cylindrospermopsis, Dolichospermum, Lyngbya, Oscillatoria, Raphidiopsis, Sphaerospermopsis*	[13,23,24,25]
	Saxitoxins	Neurotoxicity	57	*Anabaena* *Aphanizomenon, Cuspidothrix, Cylindrospermopsis,* *Dolichospermum, Fischerella, Geitlerinema, Lyngbya, Phormidium, Planktothrix, Raphidiopsis, Scytonema, Tolypothrix*	[11,13,21,26,27,28]
	Anatoxin-a	Neurotoxicity	4	*Anabaena, Aphanizomenon, Arthrospira, Cuspidothrix, Cylindrospermum, Dolichospermum, Microcoleus, Microcystis, Oscillatoria, Phormidium, Planktothrix, Pseudanabaena, Raphidiopsis, Tychonema*	[13,29,30]
	Anatoxin-a(s)	Neurotoxicity	-	*Anabaena*	[22,23]
	Lyngbyatoxins	Dermatoxicity	7	*Oscillatoria, Schizothrix, Lyngbya majuscula*	[31,32,33,34,35]
	Aplysiatoxins	Dermatoxicity	5	*Lyngbya majuscula*	[34,36]
**Lipopeptide** **s**	Antillatoxins	Neurotoxicity	2	*Lyngbya majuscula*	[37,38]
	Jamaicamides	Neurotoxicity	6		[39,40]
	Kalkitoxins	Neurotoxicity	7		[41,42]
	Barbamides	Molluscicidal	2		[43]
	Majusculamides	Cytotoxicity	4		[31,34,44,45]
	Hectochlorins	Cytotoxicity	5		[46,47]
	Curacins	Cytotoxicity	5		[48,49,50,51]
**Nonprotein amino acid**	*β*-*N*-methylamino-l-alanine	Neurotoxicity	4	*Anabaena, Calothrix, Cyanodictyon, Leptolyngbya, Limnothrix, Microcoleus, Microscystis, Myxosarcina, Nodularia, Nostoc, Oscillatoria, Phormidium, Planktothrix Pseudanabaena, Scytonema, Synechococcus, Trichodesmium*	[52,53,54,55,56]
**Lipoglycans**	Lipopolysaccharides	Endotoxicity	-	All genera of cyanobacteria	[57]

**Table 2:** The diversity of cyanotoxins in various countries.

Regions	Countries	MCs	CYNs,	NODs	STXs	ATXs	BMAA
**Asia** **(13 countries)**	China	**√**	**√**	**√**	**√**	**√**	**√**
	Turkey	**√**	**√**	**√**	**√**	**√**	
	Japan	**√**	**√**			**√**	**√**
	India and Korea	**√**			**√**	**√**	
	Qatar	**√**				**√**	**√**
	Bangladesh and Singapore	**√**			**√**		
	Israel, Saudi Arabia,	**√**	**√**				
	Thailand and Vietnam						
	Philippines	**√**					
**Africa** **(13 countries)**	Nigeria	**√**	**√**	**√**	**√**	**√**	
	South Africa	**√**		**√**			**√**
	Egypt	**√**	**√**				
	Kenya	**√**				**√**	
	Morocco	**√**			**√**		
	Algeria, Ethiopia, Ghana, Namibia, Tanzania, Tunisia, Uganda and Zimbabwe	**√**					
**North America** **(3 countries)**	Canada and the US	**√**	**√**	**√**	**√**	**√**	**√**
	Mexico	**√**	**√**		**√**		
**South America** **(5 countries)**	Brazil	**√**	**√**	**√**	**√**	**√**	
	Argentina and Uruguay	**√**	**√**				
	Chile	**√**					
	Peru						**√**
**Antarctica**	-	**√**	**√**	**√**	**√**		
**Europe** **(24 countries)**	France	**√**	**√**	**√**	**√**	**√**	**√**
	Finland, Italy, Poland and Portugal	**√**	**√**		**√**	**√**	**√**
	Germany and Spain	**√**	**√**	**√**	**√**	**√**	
	Sweden	**√**	**√**			**√**	**√**
	the UK	**√**			**√**	**√**	**√**
	Bulgaria	**√**			**√**	**√**	
	Czech, Greece and Serbia	**√**	**√**		**√**		
	Ireland	**√**	**√**			**√**	
	Denmark				**√**	**√**	
	Hungary	**√**	**√**				
	Latvia	**√**		**√**			
	Netherlands	**√**				**√**	
	Norway and Russia	**√**			**√**		
	Austria, Romania and Switzerland	**√**					
	Lebanon		**√**				
**Oceania** **(2 countries)**	Australia and New Zealand	**√**	**√**	**√**	**√**	**√**	**√**

Abbreviations: MCs: microcystins, CYNs: cylindrospermopsins, NODs: nodularins, STXs: saxitoxins, ATXs: anatoxins, BMAA: *β*-*N*-methylamino-l-alanine. “**√**” represents existence.
